# Rapid thyroid nodule growth is not a marker for well-differentiated thyroid cancer

**DOI:** 10.1186/s12957-015-0752-x

**Published:** 2015-12-18

**Authors:** Claudius Falch, Steffen Axt, Bettina Scuffi, Alfred Koenigsrainer, Andreas Kirschniak, Sven Muller

**Affiliations:** Department for Visceral, General and Transplant Surgery, Tuebingen University Hospital, Waldhoernlestrasse 22, 72076 Tuebingen, Germany; Department for General and Visceral Surgery, Marienhospital, Stuttgart, Germany

**Keywords:** Well-differentiated thyroid cancer, Thyroid nodule, Growth

## Abstract

**Background:**

Rapid growth of thyroid nodules is described as being associated with thyroid cancer. The objective of the study was to determine how the growth rate of thyroid nodules during follow-up is associated with the risk of thyroid cancer.

**Methods:**

Retrospective analysis of patients undergoing thyroid surgery for nodular disease and a repetitive preoperative ultrasound work-up of at least 6 months was done. Nodule growth was considered relevant when a volume increase >49 % was detected. Growth patterns were described as rapid for a volume increase present over 6 to 24 months.

**Results:**

Of the 297 analysed patients, 226 (76 %) displayed relevant nodule growth and 71 (24 %) no relevant growth. A rapid growth pattern was seen in 73 patients (32 %). Well-differentiated thyroid cancer was diagnosed in 33 patients (11 %; 27 papillary, 6 follicular) with a relevant nodule growth in 2 and no relevant growth in 31 patients. No rapid growth pattern was observed in any case of well-differentiated thyroid cancer. A rapid growth pattern occurred only in benign nodules (70 patients) and in 1 patient each with a lymphoma, a metastasis of a renal cell cancer and a metastasis of a gastric adenocarcinoma. Therapy with levothyroxine and/or iodine was administered to 129 patients (43 %) and was significantly inversely correlated with nodule growth (odds ratio 0.27; CI 95 % 0.14–0.53, *p* < 0.001).

**Conclusions:**

Thyroid nodule growth alone and especially a rapid growth pattern during follow-up for thyroid nodular disease is not a marker for well-differentiated thyroid cancer and should not be used as a stand-alone argument for thyroid surgery.

## Background

Thyroid nodules are frequently detected during neck ultrasound. Their incidence is described in up to 50 % of patients, depending on age and regional iodine intake [1]. Compared with this rate, the incidence of thyroid malignancy is rare [2]. However, because of the probability of malignancy in any thyroid nodule, ultrasound work-up is recommended [3]. Sonographic features suspicious for malignancy are internal calcification, hypoechogenity, centrally increased blood flow, infiltrative margins and a nodule taller than wider [4]. Thyroid nodule growth alone is not believed to be a sign of malignancy, but rapid nodule growth is described as being associated with thyroid cancer [5, 6]. The 2009 American Thyroid Association guidelines suggest that a 50 % threshold for nodule volume increase at follow-up US is a criterion for further diagnostic work-up to rule out malignancy [7]. Growth occurs over time in benign thyroid nodules in 20 to 39 % of patients, depending on whether levothyroxine and/or iodine is administered or not [8, 9]. Hence, one is regularly confronted with this problem during follow-up for thyroid nodules, and the definition of substantial nodule growth remains unclear.

Therefore, we aimed to investigate growth patterns of thyroid nodules during preoperative work-up and their correlation with well-differentiated thyroid malignancy.

## Methods

A series of 1209 consecutive patients undergoing thyroid resection at Tuebingen University Hospital from January 2005 to December 2013 was retrospectively analysed using our electronic patient database. All patients with a thyroid resection for nodular disease and a preoperative repetitive ultrasound work-up of at least 6 months were included in the analysis. The main outcome measure was the correlation of nodule growth and the final histopathologic diagnosis.

Patients with incomplete sonographic documentation of the width, depth and length of the thyroid nodule as well as complete cystic lesions were excluded. The following parameters were assessed: age, gender, uninodular or multinodular thyroid, final histopathologic diagnosis and preoperative therapy with levothyroxine and/or iodine. Furthermore, change in sonographic nodule characteristics as microcalcification, hypoechogenity and irregular nodule margins during follow-up were recorded. Fine needle aspiration biopsy (FNAB) was not performed routinely in the diagnostic work-up process for thyroid nodules and thus was not further analysed because of low availability in this series. According to the final histopathologic examination, underlying thyroid disease diagnosis was classified as benign nodular thyroid disease (uninodular or multinodular) and primary or secondary thyroid cancer. All thyroid specimens were evaluated histologically by a board-certified pathologist.

Thyroid nodule volume was retrospectively estimated according to Brauer et al. using the maximal width, depth and length of the nodule for calculation with the rotation ellipsoid formula: ^*V*^ellipsoid**π**^/6 * *D*^length^* *D*^width^* *D*^depth. Only an increase in the nodule volume >49 % was interpreted as a relevant nodule growth, while lesser volume changes were classified as no relevant nodule change [10]. Nodule growth pattern was further subclassified as rapid nodule growth if the nodule volume increased within 6 to 24 months. Data were analysed by using statistical software, SPSS, version 21.0.0.1 (SPSS Inc., Chicago, IL, USA). As statistical tests, the Fisher exact test, the chi-square test and Kruskal-Wallis test were used where appropriate. A *p* value of less than 0.05 was considered statistically significant. Multiple logistic regression analysis was performed to investigate the influence of confounders in determining independent variables for nodule growth (age, gender, type of nodularity, levothyroxine therapy, final pathology, initial thyroid nodule volume, follow-up duration) and for well-differentiated thyroid cancer (age, gender, type of nodularity, growth pattern, initial thyroid nodule volume, final thyroid nodule volume, follow-up duration, change in sonographic characteristics). The study was conducted in accordance with the ethical requirements regarding the protection of the rights and welfare of human subjects participating in medical research (Ethical Review Board of the University of Tuebingen, Germany).

## Results

Of 1209 patients with a thyroid resection, 368 patients had a repetitive preoperative ultrasound work-up of at least 6 months. A complete record of thyroid nodule dimensions was available in 297 (25 %) patients and was included in the final analysis. The incidence of thyroid malignancy in the overall population was 159 (13.2 %) out of 1209 patients.

Of the 297 patients ultimately analysed, 226 (76 %) displayed relevant nodule growth and 71 (24 %) no relevant growth. A rapid nodule growth pattern (<24 months) was seen in 73 (32 %) of the patients with relevant nodule growth, whereas the remaining 153 patients showed moderate growth (>24 months). Details and characteristics of patients according to their thyroid nodule growth patterns are displayed in Table 1. Thyroid nodule growth as sole criterion for surgery was present in 137 (46 %) patients, while local symptoms led to surgery in 58 patients (20 %) and suspicious ultrasound features in 32 (11 %) patients.

**Table 1:** Characteristics of the study population with followed-up thyroid nodules displaying a rapid nodule growth (increase in nodule volume over 50 % within 24 months), moderate nodule growth (increase in nodule volume over 50 % and over 24 months) and no relevant nodule growth

	Relevant nodule growth		No nodule growth	*p* value
	Rapid nodule growth <24 months	Moderate nodule growth >24 months		
No. of patients (*n*)	73 (%)	153 (%)	71 (24 %)	
Gender (female, *n*)	48 (66 %)^a^	115 (75 %)^a^	46 (65 %)^a^	0.317
Median age at surgery, [range]	48 [19–81]	54 [16–84]	49 [17–78]	0.476
Nodularity				
Uninodular	11 (15 %)	20 (13 %)	20 (28 %)	0.019
Multinodular	62 (85 %)	133 (87 %)	51 (72 %)	
Median follow-up duration, months [range]	13 [6–24]	41 [25–79]	24 [6–89]	
Initial mean thyroid nodule volume (cm^3^)	2.62 ± 0.96	2.3 ± 1.31	2.47 ± 1.29	0.598
Final mean thyroid nodule volume (cm^3^)	4.34 ± 1.69	5.97 ± 3.78	3.26 ± 2.65	<0.001
Benign disease (*n*)	70 (96 %)^a^	149 (97 %)^a^	41 (58 %)^a^	
Malignant disease (*n*)	3 (%)^a^	4 (%)^a^	30 (42 %)^a^	
Papillary thyroid cancer (>1 cm)	0	0	11	
Papillary microcarcinoma (<1 cm)	0	2	14	<0.001
Follicular thyroid cancer	0	1	5	
Secondary thyroid malignancy	3	1	0	
Therapy with levothyroxine and/or iodine^a^	17 (23 %)^a^	66 (43 %)^a^	46 (65 %)^a^	<0.023

^a^Displayed as percentage of the corresponding group

Thyroid malignancy occurred in 37 (12.5 %) of 297 patients, 33 of whom had a well-differentiated thyroid cancer and 4 secondary thyroid malignancies (2 with a metastasis of a renal cell carcinoma, 1 non-Hodgkin’s lymphoma and 1 metastasis of an adenocarcinoma of the stomach). No case of undifferentiated or medullary thyroid cancer occurred in the series. In two patients with well-differentiated thyroid cancer, lymphatic micrometastases were confirmed in the central compartment, while no gross lymphatic metastatic involvement was observed in any patient. Twenty cases of well-differentiated thyroid cancer were diagnosed in multinodular thyroid disease. Twelve of these were manifested in the dominant nodule on ultrasound, while 6 of the remaining 8 cases proved to be incidental papillary microcarcinoma.

The initial mean nodule volume was comparable for nodules with relevant rapid growth (2.62 ± 0.96 cm^3^), with relevant moderate growth (2.3 ± 1.31 cm^3^) and no growth (2.47 ± 1.29 cm^3^; *p* = 0.657). Mean volume increased to 4.34 ± 1.69 cm^3^ for nodules with relevant rapid growth, to 5.97 ± 3.78 cm^3^ for nodules with relevant moderate growth and to 3.26 ± 2.65 cm^3^ for those with no growth following a median follow-up period of 13 (range 6–24), 41 (range 25–79) and 24 (range 6–89) months, respectively. Patients with relevant thyroid nodule growth had significantly less frequently undergone therapy with levothyroxine and/or iodine as compared to patients with no relevant growth. In a multiple logistic regression analysis, only therapy with levothyroxine and/or iodine was significantly inversely correlated with thyroid nodule growth (odds ratio 0.28; CI 95 % 0.142–0.539, *p* < 0.001).

Well-differentiated thyroid cancer occurred significantly more often in patients with no relevant nodule growth than in patients with relevant nodule growth. A rapid growth pattern was not observed in any patient with well-differentiated thyroid cancer, but in 3 patients with secondary thyroid malignancies (1 patient each with a lymphoma, a metastasis of a renal cell cancer and a metastasis of a gastric adenocarcinoma), 13 with benign uninodular disease and 57 with benign multinodular disease. Levothyroxine and/or iodine therapy was not correlated with nodule dignity.

Thyroid nodule characteristics according to their histopathologic findings are displayed in Table 2. Multivariate analysis revealed only uninodularity (odds ratio 3.59; CI 95 % 1.36–9.52, *p* = 0.01) predictive for well-differentiated thyroid cancer. An inverse correlation was observed for nodule growth pattern, predicting well-differentiated thyroid cancer (odds ratio 0.11; CI 95 % 0.05–0.23, *p* > 0.001). Changes in sonographic nodule characteristics such as microcalcification, hypoechogenity and irregular nodule margins during follow-up were observed slightly more frequent in thyroid malignancies than in benign findings, but lack significance due to their rare appearance in multivariate analysis.

**Table 2:** Thyroid nodule characteristics according to their histopathologic findings; *p* value displayed as well-differentiated thyroid cancer vs. benign findings

	Benign	Well differentiated thyroid cancer			Secondary malignancy	Malignant total	*p* value
		Papillary	Follicular	Total			
*N*	260	27	6	33	4	37	
Nodularity							
Uninodular	36 (14 %)	12	1	13 (39 %)	2	15	0.001
Multinodular	224 (86 %)	15	5	20 (61 %)	2	22	
Initial nodule volume >2 cm^3^	185 (71 %)	10	5	15 (45 %)	2	17	0.004
Nodule volume >2 cm^3^ at last follow-up	226 (87 %)	12	6	18 (55 %)	3	21	0.001
Median months ultrasound follow-up of nodule before surgery	28 [6–89]			31 [6–53]	11 [6–26]		0.001
Sonomorphologic change in nodule pattern							
Hypoechogenity	24 (9 %)	5	1	6 (18 %)	3	9	0.237
Microcalcification	12 (4.6 %)	6	1	7 (21 %)	0	7	0.004
Taller than wide	11 (4.2 %)	3	2	5 (15 %)	2	7	0.036
Irregular nodule margins	5 (2 %)	1	0	1 (3 %)	1	2	0.553
Suppressive therapy with levothyroxine and/or iodine	109 (42 %)	17	2	19 (58 %)	1	20	0.292

## Discussion

Natural growth behaviour of thyroid nodules is controversially discussed. Reports of volume change in thyroid nodules over time vary from largely decreasing [11–13] to predominantly increasing [8, 9], depending on factors such as type of nodularity (uninodular vs. multinodular) [14], iodine deficiency [15], anatomic configuration of the nodule (cystic content) [6] and administration of therapy with levothyroxine [16]. Most recently, Durante et al. described a volume increase of asymptomatic and sonographic unsuspicious nodules in only 15 % over 5 years [17]. According to our results, they found a low incidence of thyroid cancer and no association with nodule growth. Furthermore, there is no clear consensus on how to define substantial thyroid nodule growth [3]. Brauer et al. reported a relevant interobserver variation in thyroid nodule volume measurement when the nodule volume change was reported to be below 50 %, resulting in conflicting interpretations [10]. They thus recommended that a nodule volume change of at least 49 % or more should be interpreted only as relevant nodule shrinkage or growth [10]. Thyroid nodule growth per se seems not to be a stand-alone marker for malignancy. Kim et al. reported that nodules showing more than 50 % growth during sonographic follow-up were unlikely to result in a diagnosis of malignancy, whereas malignancy was more often present when suspicious US features were found [18]. However, the revised American Thyroid Association guidelines from 2015 suggested that a 50 % threshold for nodule volume increase at follow-up is a criterion for further diagnostic work-up and repeat FNA to rule out malignancy (weak recommendation based on low quality evidence) [19]. Moreover, in commonplace practice, thyroid nodules with growth are often surgically treated to rule out cancer [20]. The results of this study show that relevant thyroid nodule growth over time defined as a volume increase of more than 50 % is present in three fourths of the patients followed. Moreover, rapid nodule growth within 24 months was seen in 30 % of all patients. Nevertheless, nodule growth was rarely associated with well-differentiated thyroid cancer, and a rapid thyroid nodule growth pattern occurred only in benign lesions, metastatic cancers and lymphoma. All data were obtained from a surgical database, meaning that data from patients still under follow-up without surgery were not included in this analysis. These results are thus not comparable to the results of plain observational studies reporting much lower thyroid nodule growth rates [18, 21]. In matters of nodule growth and well-differentiated thyroid cancer, this might even further increase the ratio of patients with relevant nodule growth and without thyroid malignancy.

Also, the majority of patients in this series were operated on because of relevant nodule growth without any further signs of malignancy. Only two of these mentioned patients subsequently displayed a papillary microcarcinoma in thyroid nodule under surveillance interpretable as an incidental finding without any further clinical relevance. On the other hand, patients with proven well-differentiated thyroid cancer showed no substantial nodule growth. These results are comparable with the findings made by Ajmal et al. that thyroid cancer is a very slow growing process that may not progress substantially over many years [22]. Of importance, no locally infiltrating or metastatic well-differentiated thyroid cancer was observed in the study population, which led us to conclude that the therapy concept was curative in all patients. Therefore, thyroid nodule growth as a stand-alone argument for malignancy should be abandoned.

Excluding the incidental findings of papillary microcarcinoma, 12 of 14 well-differentiated thyroid cancers diagnosed in a multinodular thyroid proved to be in the dominant nodule on ultrasound. This is in accordance with the findings made by Kunreuther et al., who reported that thyroid cancer was most often found in patients with multiple nodules in the dominant or largest nodule [23].

In addition, changes in sonographic nodule characteristics suspicious for malignancy during follow-up such as microcalcification, hypoechogenity and irregular nodule margins during follow-up were observed slightly more frequently in thyroid malignancies than in benign findings. However, these findings lacked statistical relevance mainly because patients with suspicious sonographic findings most likely underwent surgery initially, the majority of patients in this study had sonographically unsuspicious nodules in their baseline ultrasound and the incidence of thyroid malignancy during follow-up was too low to show a statistically significant difference. Recent observational studies have reported that sonographic nodule characteristics, and not nodule growth, are important signs in the diagnosis of malignancy [18].

Limitations of our findings are the facts that in addition to a potential bias due to its retrospective design, only patients with initially mainly unsuspicious nodules on ultrasound completed follow-up and definite histopathologic diagnoses after surgery were included. Three fourths of all patients operated during the study period were excluded from the analysis because no nodule follow-up was described or available. However, the incidence of well-differentiated thyroid cancer in the study population was comparable to that in the overall population of patients operated during the study period, indicating that this selection does not seem to have influenced cancer incidence.

Some reports have shown that FNAB during follow-up of thyroid nodules, especially in cystic lesions might have a short-term effect on nodule size and growth pattern, making interpretation questionable [24]. However, long-term effects of FNAB on nodule size seem to be neglectable [25]. With regard to the low number of preoperative FNAB and the exclusion of complete cystic lesions, we believe that the described alteration of nodule size due to the FNAB has no effect on our observed general conclusion.

Since rapid thyroid nodule growth might prove to be an undifferentiated thyroid cancer or secondary malignancy, correct preoperative diagnosis by means of, e.g. FNAB is of paramount importance as this might relevantly alter the treatment in most of these cases [26].

## Conclusions

Altogether, thyroid nodule growth taken alone during follow-up is not a marker for well-differentiated thyroid cancer and should not be used as a stand-alone argument for thyroid surgery.
